# Ubiquitination and Ubiquitin-Like Modifications as Mediators of Alternative Pre-mRNA Splicing in *Arabidopsis thaliana*

**DOI:** 10.3389/fpls.2022.869870

**Published:** 2022-05-12

**Authors:** Wei Lan, Yuhao Qiu, Yun Xu, Yalin Liu, Ying Miao

**Affiliations:** Fujian Provincial Key Laboratory of Plant Functional Biology, College of Life Sciences, Fujian Agriculture and Forestry University, Fuzhou, China

**Keywords:** ubiquitin and ubiquitin-like modification, pre-mRNA splicing, spliceosome components, plant development, stresses response

## Abstract

Alternative splicing (AS) is a common post-transcriptional regulatory process in eukaryotes. AS has an irreplaceable role during plant development and in response to environmental stress as it evokes differential expression of downstream genes or splicing factors (e.g., serine/arginine-rich proteins). Numerous studies have reported that loss of AS capacity leads to defects in plant growth and development, and induction of stress-sensitive phenotypes. A role for post-translational modification (PTM) of AS components has emerged in recent years. These modifications are capable of regulating the activity, stability, localization, interaction, and folding of spliceosomal proteins in human cells and yeast, indicating that PTMs represent another layer of AS regulation. In this review, we summarize the recent reports concerning ubiquitin and ubiquitin-like modification of spliceosome components and analyze the relationship between spliceosome and the ubiquitin/26S proteasome pathway in plants. Based on the totality of the evidence presented, we further speculate on the roles of protein ubiquitination mediated AS in plant development and environmental response.

## Introduction

**Figure 1:**
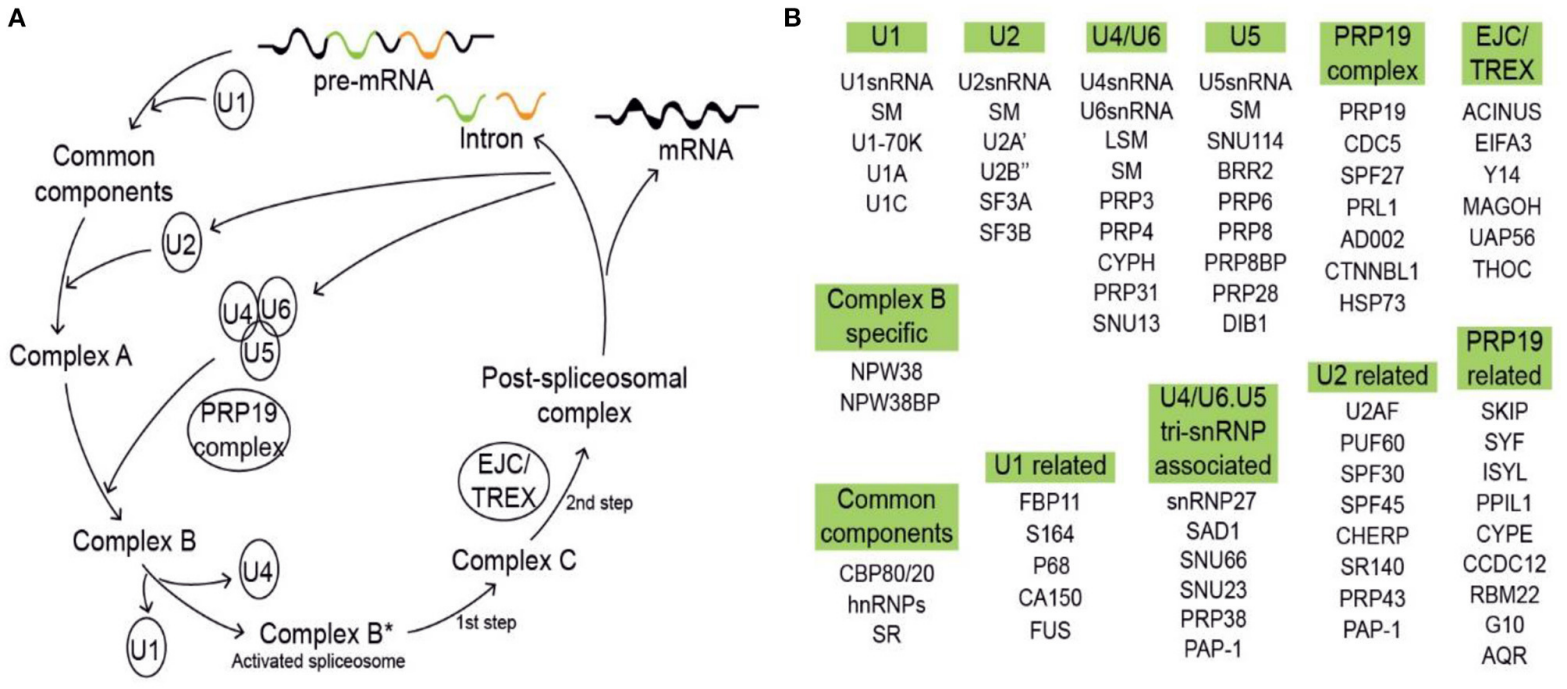
Model of pre-mRNA splicing process (drawn based on https://www.kegg.jp/pathway/map03040). **(A)** The process of pre-mRNA splicing. The initial step of splice-site recognition comprises U1 small nuclear ribonucleoprotein particle (snRNP) binding to the 5′ splice site and U2 auxiliary factor (U2AF) binding to the 3′ splice site. U2AF35, the small subunit of U2AF, binds to the intron/exon border, whereas the large subunit U2AF65 binds to a region rich in pyrimidines designated the polypyrimidine tract. Subsequently, U2 snRNP binds to the branch point, and a preformed complex of U4, U5, and U6 snRNPs is recruited to the intron. After major rearrangements and release of the U1 and U4 snRNPs, the splicing reaction takes place. **(B)** All spliceosomal components are grouped by category, including U1, U2, U4/U6, U5, PRP19 complex, EJC/TREX, U1 related, U2 related, PRP19 related, U4/U6, U5 tri-snRNP associated, and common components.

Removal of introns through pre-mRNA splicing is a key regulatory step in eukaryotic gene expression, a process which was initially characterized in viruses (Berget et al., 1977; Chow et al., 1977). During this process, introns are removed *via* the spliceosome, a high molecular weight complex that is assembled at each intron. The spliceosome is made up of five small nuclear ribonucleoprotein particles (snRNPs)—U1, U2, U4, U5, and U6 snRNPs—and over 200 additional proteins (Figure 1). The five snRNPs and their associated proteins coordinate splice-site recognition, spliceosome assembly, and catalysis of the two transesterification steps (Mckay and Johnson, 2010; Koncz et al., 2012; Reddy et al., 2013; Carvalho et al., 2016). Contained within these five snRNPs are uridine-rich small nuclear RNAs, and their core particles are formed by Sec1/Munc18 (SM) or like-SM (LSM) proteins. In addition to these snRNPs, other spliceosomal components include pre-mRNA processing factor 19 (PRP19) complex, exon junction complex (EJC)/transcription and export (TREX) complex, U1 related, U2 related, U4/U6, U5 tri-snRNP, and more (Figure 1B) (Tharun, 2009). These components are assembled on pre-mRNAs in a stepwise manner to form active spliceosomes with highly dynamic and tightly regulated conformation and composition (Figure 1A) (Tharun, 2009; Mckay and Johnson, 2010).

Following the initial discovery and characterization of spliceosomal introns, subsequent work revealed their functional roles in gene expression. In particular, eukaryotic genes can generate multiple transcripts by selective removal of specific introns, a process called alternative splicing (AS) which was initially hypothesized by Gilbert (1978). AS does not occur in isolation, and it is performed in conjunction with other pre-mRNA editing events to ensure the diversity of gene transcripts in response to various cues. The selection of splice sites in response to various cues is determined by splicing factors (SFs), which guide spliceosomal components to proper assembly on their respective splice sites (Matlin et al., 2005; Nilsen and Graveley, 2010; Wachter et al., 2012). Major SF families include serine/arginine-rich (SR) proteins and heterogeneous nuclear ribonucleoprotein particle (hnRNP) proteins (Figure 1B). These proteins bind specific pre-mRNA sequences called intronic- or exonic-splicing enhancer or suppressor sequences (Witten and Ule, 2011). Clearly, changes in SF level or activity can lead to subtle or profound effects on the splicing outcomes of downstream target genes (Stamm et al., 2005; Kalyna et al., 2006; Stauffer et al., 2010; Saltzman et al., 2011; Thomas et al., 2012). Overall, pre-mRNA splicing is a highly dynamic and tightly regulated post-transcriptional process which occurs in a stepwise manner (Wahl et al., 2009; Mckay and Johnson, 2010; Will and Luhrmann, 2011). Significant strides have been made in describing the dynamics of spliceosome machinery in recent years, yet the mechanisms by which spliceosomal proteins mediate the ordered rearrangements within the spliceosome remain elusive.

Post-translational modifications (PTMs), including phosphorylation, acetylation, O-GlcNAcylation, methylation, ubiquitination, ubiquitin-like modification, and more, work in every cellular process to reversibly adjust and fine-tune protein activity and to direct subcellular localization. PTMs play important roles in the splicing cycle and in ensuring splicing fidelity by facilitating the rearrangements of the spliceosome, controlling the timing of spliceosome rearrangements, regulating splicing factor activities, or altering the mRNP composition assembled on a particular transcript (Mckay and Johnson, 2010). Among these PTMs, ubiquitination or ubiquitin-like modification functions as a key regulator of various processes in eukaryotic cells (Smalle and Vierstra, 2004). These two forms of PTMs modify target proteins *via* covalent or non-covalent conjugation with small molecules including ubiquitin, small ubiquitin-related modifier (SUMO), and homologous to ubiquitin 1 (HUB1). These modifications can cause the target proteins to either be sequentially translocated to the proteasome for degradation, or to acquire new interactors which can change subcellular localization, affinity, and function (Smalle and Vierstra, 2004). Many proteins undergo modification in this manner, including transcription factors, various enzymes, and functional proteins, and also some spliceosome-associated proteins (Mckay and Johnson, 2010; Hu and Sun, 2016; Chanarat and Ishra, 2018; Xu, 2020). Indeed, PTMs of spliceosome components have recently emerged as major regulatory factors in yeast and human cells (Mckay and Johnson, 2010; Hu and Sun, 2016; Chanarat and Ishra, 2018; Pozzi et al., 2018; Xu, 2020; Li et al., 2021; Mulvaney et al., 2021).

In this review, we summarize recent reports about ubiquitin and ubiquitin-like modification of AS components and analyze the relationship between spliceosome and the ubiquitin (Ub)/26S proteasome pathway in plants. We further provide speculation about the mechanism of these PTMs in controlling plant development and response to environmental cues.

## The Regulation of as Through PTMs

As discussed above, PTMs are involved in the regulation of protein folding, activity, stability, localization, and interactions with other proteins/molecules through covalent or non-covalent modification of the protein by functional chemical groups, including phosphate, acetyl, methyl, carbohydrate, ubiquitin, and ubiquitin-like modifications (Deribe et al., 2010; Mckay and Johnson, 2010; Lin and Begley, 2011; Chiang and Ack, 2017). Recent evidence suggests that PTM of AS components may represent another layer of AS control. Specifically, recent reports have revealed that ubiquitin and ubiquitin-like modifications of spliceosome play a role in regulating eukaryotic pre-mRNA splicing in mammalian cells. These modifications have been shown to promote splicing by generating new surfaces for intermolecular interactions, thereby refining gene expression (Chanarat and Ishra, 2018).

### Biochemical Aspects of Ubiquitin and Ubiquitin-Like Modification

Ubiquitin and ubiquitin-like modification is a dynamic and reversible process involving the covalent attachment of a conserved 76-amino acid peptide ubiquitin (ub) to the ε-amino groups of lysine residues in target proteins. Ubiquitin addition is catalyzed by an enzymatic cascade involving an E1 ub-activating enzyme, followed by the sequential activities of E2 ub-conjugating enzyme and E3 ub ligase (Smalle and Vierstra, 2004; Lan and Miao, 2019). This process is reversible as a result of deubiquitinating enzymes (DUBs). DUBs are responsible for the removal of ubiquitin from their target proteins, rescuing them from the degradative pathway (Reyes-Turcu et al., 2009). Mono-ubiquitination has emerged to have an important signaling role which is distinct from polyubiquitination. Specifically, mono-ubiquitination of both histones and histone-associated proteins has been shown to regulate transcription, DNA repair, signal transduction, and receptor internalization (reviewed in Welchman et al., 2005). Ubiquitin and ubiquitin-like modifications have also been shown to modulate protein–protein interactions, often serving as an initiating signal for subsequent downstream phosphorylation and methylation in splicing events (Li et al., 2021; Mulvaney et al., 2021).

### Ubiquitin-Like Modification of Spliceosomal Components

**Figure 2:**
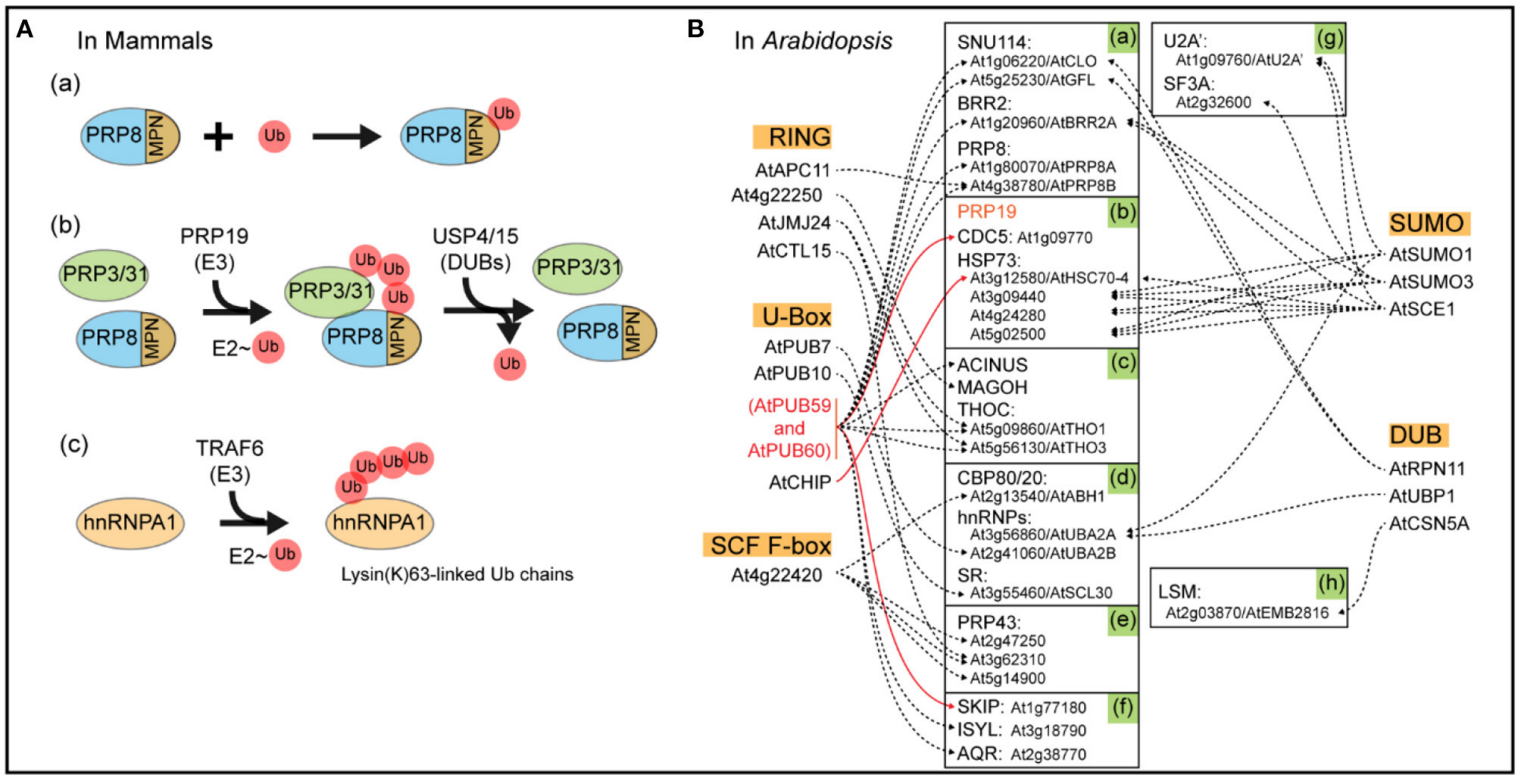
Schematic diagram of ubiquitination in mammals and *Arabidopsis*. **(A)** Ubiquitination and deubiquitination of mammalian spliceosomal components. MPN, the MPN domain, a variant of the Jab1/MPN domain found in a class of deubiquitinating enzymes, located near the C terminus of PRP8p; Ub, Ubiquitin. **(B)** The ubiquitin-associated interacting proteins. Shown in the orange box are RING E3s, U-Box E3s, SCF F-box E3s, SUMOylation components, and DUBs of *Arabidopsis* spliceosomal components (https://www.kegg.jp/pathway/ath03040). Shown in the green highlighted boxes are components of U5 (a), PRP19 complex (b), EJC/TREX (c), common components (d), U2 related (e), Prp19 related (f), U2 (g), and U4/U6 (h). The solid line denotes established interactions; the broken line denotes putative interaction. These interactome networks were downloaded from the BioGRID^4.4^ database (https://thebiogrid.org/).

Since the initial discovery of ubiquitin, a number of ubiquitin-like protein modifications (e.g., SUMO and HUB1) have been identified (Jentsch and Pyrowolakis, 2000). SUMO proteins are covalently attached to targets in a stepwise manner through a process called SUMOylation (small ubiquitin-related modifier modification). The consequences of SUMOylation are target-dependent and include altering protein activity, subcellular localization, and protection from ubiquitination (Melchior, 2000). The first study on the roles of SUMOylation in pre-mRNA splicing and mRNA metabolism is the discovery that SUMO E2-conjugating enzyme UBC9 is located in nuclear speckles. These are discrete subnuclear structures that are responsible for pre-mRNA splicing and mRNA export, often co-localizing with SC35 (Ihara et al., 2008; Spector and Lamond, 2011). In addition, SUMO proteomics of human cell lines and yeast have shown that the SR proteins, members of the hnRNP family, and many SFs are sumoylated upon exposure to stress (Golebiowski et al., 2009; Psakhye and Jentsch, 2012; Pozzi et al., 2017, 2018; Riboldi et al., 2021). Although there is limited evidence of spliceosomal SUMOylation in plants to this point, according to the public database Biological General Repository for Interaction Datasets (BioGRID) (https://thebiogrid.org/) (Altmann et al., 2020; Oughtred et al., 2021), the SUMO E2, AtSCE1 (AT3G57870), can interact with six spliceosome components, including AT1G09760/U2A' and AT1G20960/AtBRR2A (Figure 2B). In addition to AtSCE1, the two SUMOs AT4G26840/AtSUMO1 and AT5G55170/AtSUMO3 are capable of interacting with several splicing components (Figure 2B). Taken together, this *in silico* analysis suggests that SUMOylation is likely to regulate pre-mRNA splicing in plants.

In contrast to SUMO, the ubiquitin-like protein HUB1 interacts with SNU66, a component of the U4/U6 complex. Furthermore, it has been reported that the U5 tri-snRNP complex is modified by HUB1 non-covalently binding to the N terminus of SNU66, thereby modulating pre-mRNA splicing in *S. cerevisiae* (Wilkinson et al., 2004; Mishra et al., 2011; Ammon et al., 2014). HUB1 promotes splicing of introns containing non-canonical 5′-splice sites and AS of the SRC1 gene (Mishra et al., 2011). Another report showed that AtUBL5/AtHUB1 is involved in root elongation, plant development, and auxin response through pre-mRNA splicing in *Arabidopsis* (Ammon et al., 2014; Sasaki et al., 2015; Haak et al., 2017; Park et al., 2021).

### Ubiquitination and Deubiquitination of Spliceosomal Components in Mammalian Cells

Ubiquitination proceeds *via* a three-step conjugation cascades: ubiquitin becomes 'activated' when an E1 enzyme uses the energy supplied by ATP hydrolysis to generate a thioester bond between its active-site cysteine residue and the C-terminus of ubiquitin, then ubiquitin is transferred onto the active-site cysteine of an E2 enzyme. The charged E2 finally interacts with an E3 ligase that facilitates positioning and transfer of ubiquitin onto the substrate by one of several mechanisms dependent on the class of E3 enzyme (Smalle and Vierstra, 2004). These polyUb–protein conjugates are then either targeted to the 26S proteasome for degradation, or acquire new binding surfaces that alter the protein's subcellular localization, affinity, and function. Deubiquitination is a reverse reaction of ubiquitination, generating free ubiquitin from polyUb–protein conjugates *via* the action of deubiquitinating enzymes (DUBs) (Smalle and Vierstra, 2004). In *Arabidopsis* genome, there are >1,400 (or >5% of the proteome) ubiquitination and deubiquitination pathway components that can be connected to almost all biological processes (Vierstra, 2003). This includes 17 ubiquitin (Callis et al., 1990, 1995), 2 E1s, 45 E2s (Ramadan et al., 2015), >1,200 E3s (Gagne et al., 2002; Capron et al., 2003; Stone et al., 2005; Trujillo, 2018), 53 26S proteasome components (Fu et al., 1999; Kurepa and Smalle, 2008), and 45 DUBs (Yan et al., 2000; Isono and Nagel, 2014).

Initial reports on ubiquitin's function in pre-mRNA splicing are only ~10 years old, and subsequent work has revealed that ubiquitination controls a large number of diverse cellular processes. One study reported that a variant of the Jab1/MPN domain near the C-terminus of spliceosome component PRP8p directly binds to ubiquitin with an affinity comparable to other known ubiquitin-binding domains. This was one of the first reports suggesting that ubiquitin functionally regulates pre-mRNA splicing machinery (Figure 2A-a) (Bellare et al., 2006). In another study, the spliceosomal E3 ubiquitin ligase PRP19 recruits K63-linked ubiquitin chain to PRP3/PRP31 proteins, thereby stabilizing the association between PRP3/31 and the core spliceosomal protein PRP8 in the pre-catalytic mammalian spliceosome (Bellare et al., 2008; Song et al., 2010). PRP3 is a core SF of the U4/U6. Both the U5 tri-snRNP complex and PRP31 are components of the U4 snRNP. These associations need to be weakened in order to initiate catalytic activity of the spliceosome, which takes place through the removal of the ubiquitin chain from PRP3 by the deubiquitinating enzyme USP4 and from PRP31 by USP15 (Figure 2A-b) (Song et al., 2010; Park et al., 2016; Das et al., 2017). The TRAF6 E3 ligase regulates pre-mRNA splicing through ubiquitination of hnRNPA1, which leads to AS of *ARHGAP1*, an inhibitor of the GTP-binding Rho family protein CDC42. One report showed that this process led to activation of CDC42 and hematopoietic defects in TRAF6-expressing hematopoietic stem/progenitor cells (Figure 2A-c) (Fang et al., 2017).

### Association Between Spliceosome and Ubiquitination Pathway During *Arabidopsis* Development

According to KEGG pathway analysis in *Arabidopsis thaliana* (https://www.kegg.jp/pathway/ath03040), there are more than 175 *Arabidopsis* spliceosomal protein components. Potential interacting proteins of the *Arabidopsis* spliceosomal components are then determined using BioGRID^4.4^ database analysis (Figure 2B) (Altmann et al., 2020; Oughtred et al., 2021). PRP19 is unique in that it is a component of the spliceosome and also one of the U-Box E3 ubiquitin ligases. In *Arabidopsis*, there are two homologies of PRP19—AtPUB59 (AT1G04510, also called MAC3A) and AtPUB60 (AT2G33340, also called MAC3B). According to BioGRID^4.4^, 11 spliceosome-associated proteins may interact with AtPUB59 and AtPUB60, including SUN114, BRR2, PRP8, CDC5, ACINUS, THOC, ISYL, and AQR (Figure 2B-a,f). At present, there is no evidence showing that AtPUB59 and AtPUB60 are involved in the ubiquitination of AtPRP3 and AtPRP31 in mammalian cells, but they are components of the multi-protein assembly MOS4-associated complex. This complex includes AtMOS4, AtCDC5, AtPRL1, AtPUB59, and AtPUB60 and is required for basal and R protein-mediated resistance in *Arabidopsis* (Monaghan et al., 2009). *Arabidopsis* MAC3A and MAC3B act redundantly in microRNA biogenesis, which targets many genes required for proper development (Li et al., 2018). Moreover, AtPUB59 and AtPUB60 physically and genetically interact with AtSKIP, mediating the AS of about 50% of expressed genes in *Arabidopsis* genome (Figure 2B-f) (Li et al., 2019). AtSKIP has previously been reported to be involved in 26S proteasome-mediated degradation (Li et al., 2016), affecting both the circadian clock and salt tolerance in plants (Wang et al., 2012; Feng et al., 2015).

With the exception of AtPUB59 and AtPUB60, there are 4 RING-type E3 ligases, 3 U-Box type E3 ligases, 1 SCF F-box E3 ligases, 3 DUBs, and 25 26S proteasome components which interact with spliceosomal components (Figure 2B), assuming that spliceosomal components may be ubiquitinated by E3 ligases and then degraded by 26S proteasome or deubiquitinated by DUBs. Although there is no direct biochemical evidence, *in silico* analysis of proteomic datasets indicates that the components of U5, ribosomal protein S5/Elongation factor G/III/V family proteins AtCLO and AtGFL, may interact with AtPUB59, AtPUB60 ligases, and multi-ubiquitin chain binding protein AtRPN11, a deubiquitinating enzyme. Collectively, this suggests that AtCLO and AtGFL may be ubiquitinated by AtPUB59 and AtPUB60 and deubiquitinated by AtRPN11 (Figure 2B-a).

AtBRR2 is a component of the spliceosome and highly conserved in eukaryotes. *Arabidopsis* BRR2a is ubiquitously expressed in all tissues and involved in the processing of gene transcripts which regulate flowering time, most notably *FLC* (Mahrez et al., 2016). It may be that *FLC* interacts with Ub-protein ligases AtPUB59 and AtPUB60, and SUMO ligase AtSCE1 (Figure 2B-a), which portends that AtBRR2A may be ubiquitinated and sumoylated. Furthermore, this implies that antagonism may exist between the effect of these two modifications on the targets. We have further observed that AtHSC70-4, a component of PRP19 complex, also interacts with Ub-protein ligase (AtCHIP) and SUMO ligase (AtSCE1) (Figure 2B-b).

The C-terminus of heat shock protein (HSP) 70-interacting protein (CHIP), a chaperone-dependent and U-box-containing E3 ligase, is a key enzyme involved in protein quality control by recognizing misfolded proteins through its interacting chaperones and then directing their degradation. In plants, CHIP plays an important role in regulating responses to a broad spectrum of biotic and abiotic stresses. CHIP protects chloroplasts by coordinating protein quality control both outside and inside the photosynthetic organelles and also by modulating the activity of protein phosphatase 2A, a crucial component in plant signaling networks, including abscisic acid signaling (Zhang et al., 2021). Furthermore, the interaction between AtHSC70-4 and AtCHIP is not under the precondition that AtCHIP ubiquitinates AtHSC70-4, but is predicated on the basis that AtHSC70-4 and AtCHIP together mediate plastid precursor degradation (Lee et al., 2009). Therefore, the interaction between AtHSC70-4 and AtSCE1 mediates plastid precursor SUMOylation (Watson et al., 2021). There are multiple examples of Ub-protein ligases interacting with the same substrate, including anaphase-promoting complex AtAPC11, AtPUB59, and AtPUB60 interacting with AtPRP8B and AtJMJ24, AtPUB59, and AtPUB60 binding a nuclear matrix protein-like protein complex THOC (AtTHO1 and AtTHO3) and more. Indeed, as shown in Figure 2B-a,c, various Ub-protein ligases may support different kinds of ubiquitin chain modification for the same substrate.

In plants, the anaphase-promoting complex/cyclosome (APC/C) consists of at least 11 core subunits, each of which is encoded by a single gene, with exception of APC3, which is encoded by two genes, APC3a/CDC27a and APC3b/CDC27b/HOBBIT. The *Arabidopsis* APC/C activator cell division cycle 20.1(CDC20.1) is required for bivalent alignment and chromosome segregation during meiosis (Niu et al., 2015), suggesting that APC/C subunits may also have meiotic functions in plants. It was recently shown that *Arabidopsis* APC8 is necessary for male meiosis of plants (Liu et al., 2019). In addition to the APC complexes, THO/TREX complexes have been implicated in the transport of mRNA precursors in mammals. Mutants of THO3/TEX1, THO1, and THO6 in *Arabidopsis* accumulate reduced amounts of small interfering RNA, suggesting a role for the putative *Arabidopsis* THO/TREX in small interfering RNA biosynthesis and trafficking (Tao et al., 2016; Doll et al., 2018).

Taken together, the potential interaction between the spliceosome and ubiquitination or SUMOylation pathway in *Arabidopsis* indicates that these PTMs play an important role in controlling spliceosomal components (Figure 2B). As such, spliceosome PTMs are important for regulating RNA biogenesis, trafficking precursors of small RNAs (including microRNAs), and controlling protein quality, leading to proper plant development and adaptation to environmental stress.

### Potential Ubiquitin Modification Sites in *Arabidopsis* Spliceosomal Components

The rise of modern proteomics has uncovered a vast array of ways in which proteins can be post-translationally modified. Here, we summarize the potential ubiquitination sites of *Arabidopsis* spliceosomal components based on the Plant PTM Viewer database (https://www.psb.ugent.be/webtools/ptm-viewer/index.php) (Table 1), which provides innovative tools to analyze the potential role of PTMs in regulating specific proteins or in a broader systems biology context (Wagner et al., 2011; Willems et al., 2019).

**Table 1:** Potential ubiquitination sites in *Arabidopsis* spliceosomal components.

**snRNPs**	**Protein name**	**Tair ID**	**Ubiquitin modification sites (Predicted from Plant PTM Viewer)**
U1	U1-70K	AT3G50670	K153, K155, K165, K193, K197, K203, K402
U1 related	CA150	AT3G19840	K226, K829
	FUS	AT5G58470	K363, K371
U2	SF3A	AT1G14640	K722, K733
		AT2G32600	K94, K119
	SF3B	AT4G21660	K212
		AT5G64270	K94, K128
U2 related	U2AF	AT1G27650	K13
		AT5G42820	K13
	SPF30	AT2G02570	K173, K291
	SPF45	AT1G30480	K127
	SR140	AT5G25060	K130, K852
U4/U6	LSM	AT2G03870	K59
		AT2G43810	K13
		AT5G27720	K48, K67, K78, K83
	CYPH	AT2G38730	K175
U5	PRP6	AT4G03430	K402
U4/U6. U5 tri-snRNP	SNU66	AT5G16780	K5, K7, K76, K144, K171, K353, K459
	SNU23	AT3G05760	K24, K133, K134, K136, K152, K161
Prp19 complex	CDC5	AT1G09770	K173, K443
	SPF27	AT3G18165	K182
	AD002	AT3G13200	K29, K42, K56, K76, K209
	HSP73	AT1G16030	K512
		AT1G56410	K74, K252, K506, K513
		AT3G09440	K163, K252, K256, K257, K457, K506, K513, K563,
		AT3G12580	K252, K457, K506, K513
		AT4G24280	K131, K146, K152, K161, K198, K523, K598
		AT5G02490	K74, K252, K457, K506, K513, K563
		AT5G02500	K74, K163, K252, K254, K256, K334, K457, K506, K513
		AT5G49910	K131, K146, K161, K198
Complex B	NPW38	AT2G41020	K50, K67
Prp19 related	SKIP	AT1G77180	K205, K341, K526, K529
	SYF	AT2G16860	K45, K96, K259, K273, K274
		AT5G28740	K903
	ISYL	AT3G18790	K7, K80, K145, K179
	PPIL1	AT2G36130	K56, K138, K156
	CCDC12	AT3G05070	K11, K16, K20, K32, K48, K108, K109, K112, K130
	RBM22	AT1G07360	K302
EJC/TREX	ACINUS	AT4G39680	K453
	EIFA3	AT3G19760	K57
	Y14	AT1G51510	K53, K64
	UAP56	AT5G11170	K54, K184
		AT5G11200	K54, K184
	THOC	AT1G66260	K166
		AT5G02530	K14, K166
		AT5G09860	K14, K152
		AT5G37720	K14, K152
		AT5G59950	K15, K128
Common component	SR	AT1G02840	K175
		AT1G23860	K71
		AT3G49430	K63, K178
		AT5G18810	K94, K106

Although ubiquitination of plant spliceosomal components has not been well reported, a number of putative ubiquitination sites exist on lysine residue in these proteins, particularly the AtPRP19 complex and AtHSP73 proteins in *Arabidopsis* (Table 1) (Makarova et al., 2004; Grote et al., 2010; Slane et al., 2020). HSP70s are critical for maintaining cell viability in response to a large variety of cellular stresses, including high temperature, nutrient starvation, osmotic shock, oxidative stress, and DNA damage (Rosenzweig et al., 2019). In fact, recent work has uncovered a vast array of PTMs on HSP70 family proteins including phosphorylation, acetylation, ubiquitination, AMPylation, and ADP-ribosylation (Nitika et al., 2020). In addition, two interacting proteins of AtPUB59 and AtPUB60, AtCDC5 and AtSKIP, have potential ubiquitination sites, further suggesting that AtCDC5 and AtSKIP may be ubiquitinated by AtPUB59 and AtPUB60 or other Ub-protein ligases. These potential ubiquitination sites in spliceosomal components afford researchers with enormous opportunities to uncover novel post-translational regulatory mechanisms of spliceosome activity.

## As ff Ub-Protein Ligase mRNAs

In addition to those spliceosome components discussed above, there is another aspect of pre-mRNA splicing which can potentially be regulated by ubiquitination modification. Recently, it was shown that salt-responsive alternatively spliced gene 1 (SRAS1), a RING-type E3s, undergoes salt-responsive AS generating two splicing variants—SRAS1.1 (the full-length SRAS1) and SRAS1.2 (a small-sized protein lacking 59 amino acids in the C-terminal RING finger domain). Interestingly, these variants exhibit reciprocal responses to salt stress—SRAS1.1 expression is enhanced, while SRAS1.2 is suppressed. Moreover, SRAS1.1 promotes degradation of the COP9 signalosome 5A, which plays an important role in plant development and stress responses *via* the 26S proteasome (Gusmaroli et al., 2004; Zhou et al., 2021). In contrast, SRAS1.2 preserves the COP9 signalosome 5A by competing with SRAS1.1 on the same binding site (Zhou et al., 2021). This finding underscores the complexity of the interaction between ubiquitin-related PTMs and AS and suggests mutual regulation between these processes.

**Figure 3:**
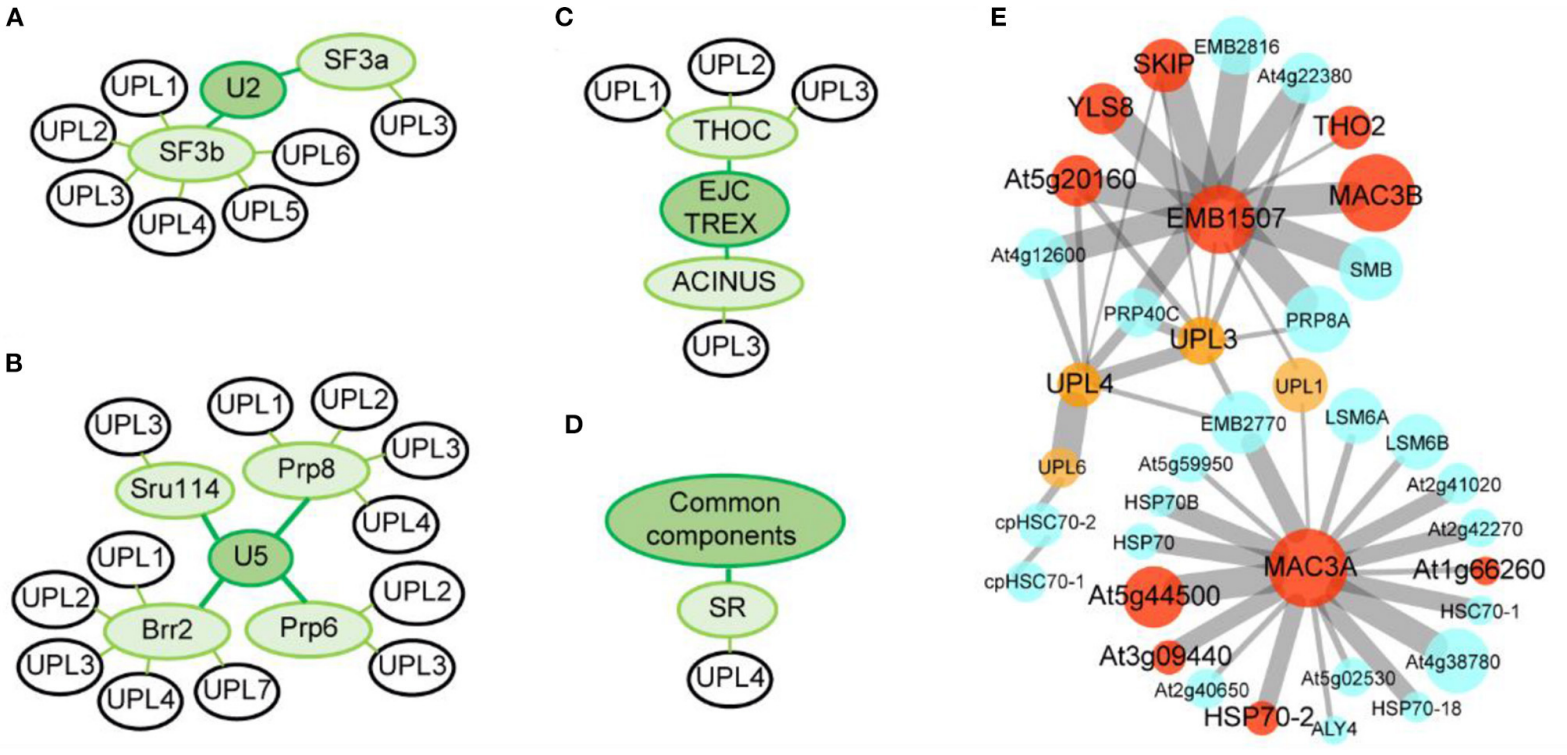
Relationship between spliceosomal components and HECT E3s. **(A–D)** Co-expression networks of HECT E3s family members with spliceosomal components. Green colored circles represent established spliceosomal components, and black-outlined circles represent HECT E3s. Proteomic analysis of spliceosomal components in the *upl3* and the *upl4/upl3* double mutants (Lan et al., 2022; unpublished data). **(E)** Red circles show upregulated genes, yellow circles show HECT E3s, while blue circles represent no change or undetected genes. Edge width indicates the strength of the relation between two proteins based on STRING database, (https://string-db.org/cgi/input?sessionId=HOnM9xTdLFXCandinput_page_active_form=multiple_identifiers), and editing through Cytoscape software.

We have recently observed that *Arabidopsis* HECT-type E3 ligases are co-expressed with spliceosomal components. To perform this analysis, we downloaded the top 200 co-expressed genes of spliceosomal components from ATTED-II (https://atted.jp/), including SF3A, SF3B, PRP6, PRP8, SNU114, BRR2, THOC, ACINUS, and SR (**f**) (Figures 3A–D). Interestingly, Furniss et al. found that the *upl3* mutant shows impairment in transcriptome reprogramming following salicylic acid treatment and failed to establish immunity against a hemi-biotrophic pathogen (Downes et al., 2003; Furniss et al., 2018). Based on this finding, it is reasonable to suggest that this impaired reprogramming may be related to AS. In fact, our comparative proteomic analysis of the HECT E3 *upl4/upl3* double mutant relative to wild type shows that most spliceosomal components are upregulated in *upl4/upl3* double mutant with developmental defects and stress-sensitive phenotypes (Figure 3E), suggesting that there is a potential relationship between pre-mRNA splicing and HECT E3s.

## The Important Roles of as for Plant Development and Stress Responses

**Figure 4:**
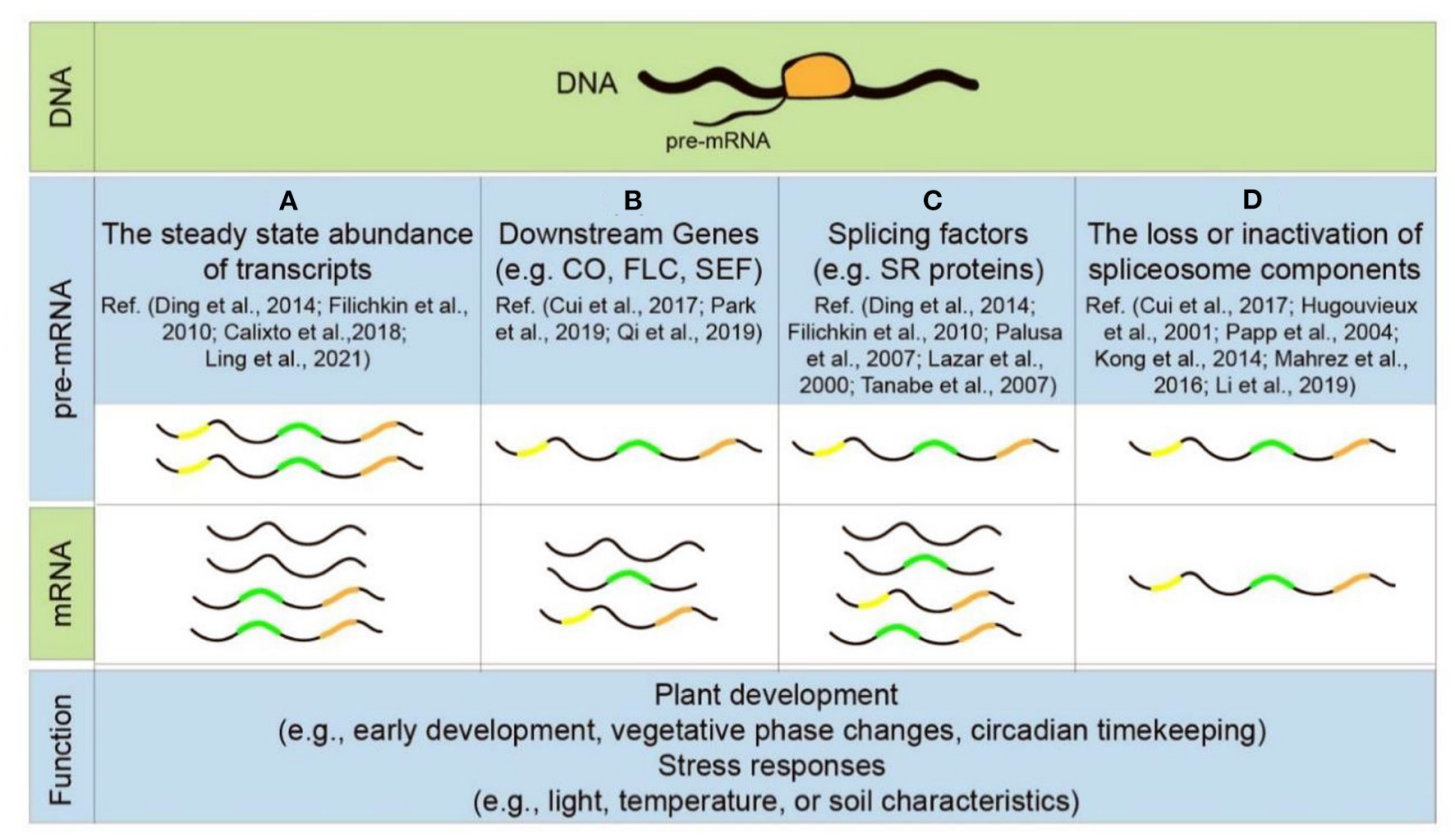
Patterns of alternative splicing during plant development and stress response. Each step of pre-mRNA splicing influences plant growth and adaptation to environments, such as the steady-state abundance of transcripts **(A)**, the alternative splicing of downstream genes **(B)**, splicing factors **(C)**, and the loss or inactivation of spliceosomal components **(D)**.

It is now fully established that at least 95% of multi-exon genes in mammals undergo some form of AS (Pan et al., 2008; Wang et al., 2008), while only 40–70% of genes undergo AS in plants (Chamala et al., 2015; Zhu et al., 2017; Chaudhary et al., 2019). AS not only affects the abundance of mature mRNAs, but also intricately regulates transcription, translation, and downstream mRNA metabolic events including mRNA export and turnover in eukaryotes (Black, 2003; Moore and Proudfoot, 2009). In addition, splicing variants produce truncated proteins that may have different subcellular localization and/or functions (Samach et al., 2011; Shin et al., 2015; Wang et al., 2015; Ghelli et al., 2018). Thus, AS fulfills many vital biological functions in plants, including developmental regulation and adaptation to stress environments (Figure 4).

Specifically, AS has been implicated in the response to environmental cues in plants, including major deviations in ambient light, temperature, and soil characteristics (e.g., water/salt content) under normal or optimal conditions (Laloum et al., 2018; Punzo et al., 2020). It not only alters the steady-state abundance of many stress-induced gene transcripts but also evokes changes in AS patterns of these transcripts (Figures 4A,B) (Filichkin et al., 2010; Ding et al., 2014; Haak et al., 2017; Calixto et al., 2018; Ling et al., 2021). For example, about ~49% of all intron-containing genes are alternatively spliced under salt stress, 10% of which experience significant differential AS, a process significantly increased under salt stress conditions (Ding et al., 2014; Haak et al., 2017). It is also worth noting that at least 95 transcripts of SR proteins, produced from only 15 genes (Palusa et al., 2007), undergo AS themselves in response to stress (Figure 4C) (Lazar and Goodman, 2000; Palusa et al., 2007; Tanabe et al., 2007; Filichkin et al., 2010; Ding et al., 2014). Another classic example is AtSR45a protein, a homolog of metazoan TRA-2. There are six types of mRNA variants (*AtSR45a-1a-e* and *AtSR45a-2*) generated by alternative selection of transcriptional initiation sites and AS processing of introns in *AtSR45a* pre-mRNA. *AtSR45a-1a* and *AtSR45a-2* expression is greatly increased by high-light irradiation (Tanabe et al., 2007; Carvalho et al., 2016). In addition, the loss or inactivation of spliceosomal components directly leads to plant growth defects and stress-sensitive phenotypes such as *abh1, brr2a*, and *skip* mutants (Figure 4D) (Hugouvieux et al., 2001; Papp et al., 2004; Kong et al., 2014; Mahrez et al., 2016; Cui et al., 2017; Li et al., 2019).

As an important factor in post-transcriptional gene regulation, AS is intimately involved in the regulation of developmental processes such as seedling development, juvenile vegetative phase, adult vegetative phase, senescence, flowering, and circadian timekeeping (Cui et al., 2017; Gil et al., 2017; Szakonyi and Duque, 2018; Bai et al., 2019; Park et al., 2019; Qi et al., 2019). For example, the floral activator CONSTANS (CO), which plays a crucial role in photoperiodic flowering (Gil et al., 2017), undergoes AS producing two isoforms—the full-size physiologically functional COα and the C-terminally truncated COβ (Qi et al., 2019). COβ acts as a competitive inhibitor of COα by forming non-functional heterodimers which have a significantly reduced DNA binding capability compared to the COα-COα homodimers (Qi et al., 2019). AS of polyadenylated ETHYLENE RESPONSE FACTOR4 (ERF4), a positive regulator of leaf senescence, results in two ERF4 isoforms: ERF4-R which contains the EAR-motif, and ERF4-A which lacks it (Lyons et al., 2013). ERF4-A acts as a transcriptional activator and ERF4-R as a repressor of their direct target gene CATALASE3, controlling the concentrations of reactive oxygen species (ROS) in cells and regulating leaf senescence (Riester et al., 2019). A minor spliceosome component U11-48 K, which is indispensable for correct splicing of U12 introns, is required for normal plant development (Gault et al., 2017; Bai et al., 2019).

Taken together, it is clear that pre-mRNA splicing plays a pivotal role in plant development and adaptation to stress environments. As such, the spliceosome activity in plants is indispensable during plant growth and stress responses.

## Conclusions and Perspective

The AS fulfills vital biological functions, including control of plant development and adaptation to stress environments. AS processes not only affect the abundance and quality of mature mRNAs in eukaryotes, but are also intimately involved in transcription, translation, and downstream mRNA metabolic events including mRNA export and turnover. PTMs of spliceosomal components and SFs provide an effective platform for mechanistic fine-tuning of the pre-mRNA splicing process. Both the spliceosomal and ubiquitination systems contain a large number of components and are ubiquitous in eukaryotes, which indicate the possibility of co-evolutionary interaction between them.

Development of new and more sensitive mass spectroscopic techniques and artificial intelligence prediction, combined with recent structures of spliceosomal complexes, will allow researchers to address challenging questions regarding splicing regulation. Furthermore, the evolution of proteomics and systems biology presents an opportunity to address complex issues concerning splicing reactions and molecular machinery. Multi-omics analysis combined with classical genetic methods or CRISPR editing will provide even more genome-wide evidence to reveal the interaction between AS and PTMs, and their roles during plant development and stress response. Finally, structural biology, phase separation, and cryo-EM remain the most effective approaches to elucidate splicing machinery dynamics and timing of AS and their PTMs during plant development and in response to environmental cues.

## Author Contributions

WL, YX, and YL are involved in writing—original draft preparation. WL, YQ, and YM are involved in writing—reviewing and editing. YM supervised the study and involved in project administration. YM and WL took a leading role in funding acquisition. All authors have read and agreed to the published version of the manuscript.

## Funding

This research was supported by the Scientific Research Foundation of Graduate School of Fujian Agriculture and Forestry University to WL and the National Natural Science Foundation of China (No. 324-1122yb049).

## Conflict of Interest

The authors declare that the research was conducted in the absence of any commercial or financial relationships that could be construed as a potential conflict of interest.

## Publisher's Note

All claims expressed in this article are solely those of the authors and do not necessarily represent those of their affiliated organizations, or those of the publisher, the editors and the reviewers. Any product that may be evaluated in this article, or claim that may be made by its manufacturer, is not guaranteed or endorsed by the publisher.
